# Ophthalmologic Baseline Characteristics and 2-Year Ophthalmologic Safety Profile of Pramipexole IR Compared with Ropinirole IR in Patients with Early Parkinson's Disease

**DOI:** 10.1155/2016/8298503

**Published:** 2016-12-18

**Authors:** William Seiple, Danna Jennings, Richard B. Rosen, Leona Borchert, Lee Canale, Nora Fagan, Mark Forrest Gordon

**Affiliations:** ^1^Lighthouse Guild, Arlene R. Gordon Research Institute, New York, NY, USA; ^2^Department of Ophthalmology, NYU School of Medicine, New York, NY, USA; ^3^Institute of Vision, Aging in Vision and Action Lab, CNRS-INSERM, University Pierre & Marie Curie, Paris, France; ^4^Jesse Brown VA Medical Center, Chicago, IL, USA; ^5^Institute for Neurodegenerative Disorders, Suite 8B, 60 Temple Street, New Haven, CT 06510, USA; ^6^New York Eye & Ear Infirmary and Icahn School of Medicine of Mount Sinai, 310 East 14th Street, New York, NY 10003, USA; ^7^Boehringer Ingelheim Pharmaceuticals, Inc., 900 Ridgebury Road, Ridgefield, CT 06877, USA

## Abstract

*Background.* Parkinson's disease (PD) progressively affects dopaminergic neurotransmission and may affect retinal dopaminergic functions and structures.* Objective.* This 2-year randomized, open-label, parallel-group, flexible-dose study, NCT00144300, evaluated ophthalmologic safety profiles of immediate-release (IR) pramipexole and ropinirole in patients with early idiopathic PD with ≤6 months' prior dopamine agonist exposure and without preexisting major eye disorders.* Methods.* Patients received labeled IR regimens of pramipexole (*n* = 121) or ropinirole (*n* = 125) for 2 years. Comprehensive ophthalmologic assessments (COA) included corrected acuity, Roth 28-color test, slit-lamp biomicroscopy, intraocular pressure, computerized visual field test, fundus photography, and electroretinography.* Results.* At baseline, we observed retinal pigmentary epithelium (RPE) hypopigmentation not previously reported in PD patients. The estimated relative risk of 2-year COA worsening with pramipexole versus ropinirole was 1.07 (95% CI: 0.71–1.60). Mean changes from baseline in Unified Parkinson's Disease Rating System parts II+III total scores (pramipexole: 1 year, −4.1 ± 8.9, and 2 years, −0.7 ± 10.1, and ropinirole: 1 year, −3.7 ± 8.2, and 2 years, −1.7 ± 10.5) and Hoehn–Yahr stage distribution showed therapeutic effects on PD symptoms. Safety profiles were consistent with labeling.* Conclusions.* The risk of retinal deterioration did not differ in early idiopathic PD patients receiving pramipexole versus ropinirole. RPE hypopigmentation at baseline was not previously reported in this population. This trial is registered with NCT00144300.

## 1. Introduction

Ocular manifestations of Parkinson's disease (PD) include visual field defects, electroretinographic changes, defective color vision and motion perception, impaired acuity (letters correct), and/or visual hallucinations [1, 2], possibly reflecting retinal dopaminergic defects and morphologic changes [3–5]. Ophthalmologic findings in early PD and their changes during progression and therapy are understudied.

Therapy options for PD motor symptoms include dopamine agonists alone in early PD [6] or adjunctively with levodopa in advanced PD [6, 7]. Pramipexole and ropinirole are nonergot dopamine agonists indicated for idiopathic PD signs/symptoms [6, 7]. Other therapies include monoamine oxidase inhibitors, levodopa, amantadine, or anticholinergics [8].

Retinal safety of dopamine agonists required reinvestigation in humans because albino rat studies showed retinal degeneration with ropinirole, pramipexole, and rotigotine; this effect was not seen in albino mice or pigmented rats, monkeys, or pigs [9–11] but involved the pan-vertebrate process of disk shedding. Few full peer-reviewed papers exist on the potential effects of pramipexole on the human retina. The US Food and Drug Administration (FDA) required a postmarketing study on eye safety of pramipexole in humans with PD, which supported a labeling revision that remains in the current label [9].

This 2-year study (NCT00144300) compared ophthalmologic safety of immediate-release (IR) pramipexole and ropinirole in early idiopathic PD, adapting a comprehensive ophthalmologic assessment (COA) previously developed for vigabatrin [12, 13].

To our knowledge, to date, this is the largest cohort of early PD subjects undergoing a prospective and complete visual assessment.

## 2. Objectives

*Primary.* The primary objective is to determine the presence or absence of retinal change or other ophthalmologic deterioration from baseline to 2 years of pramipexole compared with ropinirole in subjects with idiopathic PD. 

*Secondary.* The secondary objective is to assess and monitor safety profile and tolerability of pramipexole versus ropinirole in PD and to assess progression of PD during 2 years' treatment. 

*Post Hoc Exploratory.* Post hoc exploratory objective is to analyse baseline ophthalmologic parameters in relation to age, sex, PD stage, and PD duration and to model their 2-year longitudinal changes with respect to age, sex, PD severity, duration, and treatment effects.

## 3. Methods

### 3.1. Study Design, Ethics, and Structure

This open-label, randomized, flexible-dose, active-controlled, parallel-group, phase IV study (NCT00144300) included 21 neurology and 19 ophthalmology sites in the United States and was compliant with Declaration of Helsinki and Good Clinical Practice. The study was approved by Schulman Associates Institutional Review Board (IRB; Cincinnati, OH), Western Institutional Review Board (Olympia, WA), and/or local sites' IRBs (See Acknowledgments). All patients signed informed consent.

Electroretinography (ERG), visual field, Roth 28, and fundus photography were evaluated by central reading centers masked to treatment.

### 3.2. Inclusion and Exclusion Criteria

Eligible patients were ≥30 years old with idiopathic PD of ≤7 years' duration, with modified Hoehn–Yahr stages I–III, and with ≤6 months' cumulative lifetime dopamine agonist exposure. Exclusion criteria included nonidiopathic PD, prior stereotactic brain surgery, and existing eye abnormalities (retinopathy, dense cataracts, and glaucoma; best-corrected visual acuity [BCVA] <20/40; refractive error exceeding −6 diopters spherical; abnormal baseline ERG; eye trauma sequelae; and inability to dilate pupils). Medical history exclusions included potentially retinotoxic drug exposure within 12 months, diabetes, albinism/albinoidism, malignant melanoma, symptomatic orthostatic hypotension, current pregnancy/lactation, alcohol abuse/dependence as defined by the* Diagnostic and Statistical Manual of Mental Disorders* IV, or any other condition that could impair participation, increase risk, or confound interpretation. Baseline Mini-Mental State Examination (MMSE) scores ≤24 were exclusionary.

### 3.3. Treatment

Patients were randomized 1 : 1 to receive branded pramipexole dihydrochloride (Mirapex® IR, Boehringer Ingelheim, Ridgefield, CT) or ropinirole hydrochloride (Requip® IR, GlaxoSmithKline, Research Triangle Park, NC) through retail pharmacies. Ophthalmologists, central reading centers, the Expert Panel, and sponsor's in-house team remained masked to treatment allocation, although site investigators and subjects were aware.

Patients on prestudy dopamine agonists underwent taper-off and 14-day washout before randomization. Study drug (pramipexole or ropinirole), taken every 8 hours orally, was titrated over 13 weeks to final daily doses of 0.375–4.5 mg pramipexole or 0.75–24.0 mg ropinirole, then maintained at maximal tolerated dose for 2 years. Investigators could add levodopa, but not other dopamine agonists, during only the maintenance phase if dose escalation did not control symptoms. Amantadine, anticholinergics, or domperidone were allowed concomitantly.

### 3.4. Evaluations

#### 3.4.1. Parkinson's Disease Staging

The modified Hoehn–Yahr scale [14] and Unified Parkinson's Disease Rating Scale [15] parts II and III (UPDRS II+III) were administered at screening/baseline and after 1 year and 2 years of treatment. Baseline clinical assessments were performed off the designated study medication. Posttreatment assessments were performed on the study medication.

#### 3.4.2. Ocular Status and Retinal Function

Board-certified or eligible retinal ophthalmologists evaluated corrected acuity, ocular status, and retinal function at baseline and at 6, 12, and 24 months. Early Treatment Diabetic Retinopathy Study (ETDRS) acuity was determined at 4 meters for each eye and analyzed as “number of letters correct.” Spherical and cylindrical refraction was measured.

Computerized visual field testing used the Zeiss-Humphrey Visual Field Analyzer with white-on-white, 30-2 SITA-standard threshold strategy with age-corrected normal comparisons.

Standard ERG (ISCEV Protocol) was measured for* amplitudes* and* implicit times*: dim flash dark-adapted b-wave; bright flash dark-adapted response, a- and b-wave; dark-adapted oscillatory potentials (OPs)—sum of amplitudes of all individual OP wavelets; light-adapted 30 HZ cone flicker b-waves; and light-adapted single flash a- and b-wave. Roth 28-color test (R 28) axis and error score were determined for each eye.

Clinical ophthalmic exams included intraocular pressure (IOP); eye position; motility: full versions, smooth pursuit, and nystagmus; pupil: consensuality, direct response to light, and relative afferent pupillary defect; lids; conjunctiva; cornea; iris; and lens.

Bilateral dilated vitreous and retinal fundus (BDVRF) exam evaluated vitreous body, retinal vessels, optic disc, cup/disc ratio (horizontal and vertical), presence of macular degeneration, drusen, retinal edema or whitening, lipid exudates, retinal hemorrhage, retinal pigmentary epithelium (RPE) alteration, detachment or sensory retinal detachment, and optic nerve head abnormalities.

Fundus photographic slides were evaluated for overall impression in the ETDRS-standard 7 designated fields for retinal hemorrhage or microaneurysm, hard exudates, retinal edema or whitening, RPE hyperpigmentation, or RPE hypopigmentation/drusen/pigment epithelial detachment.

Continuous data were dichotomized by the following thresholds from the authors' clinical experience: for IOP, a clinically established value of 21 mmHg was regarded as upper bound of normal. Similar transformations were done for acuity (<20/25, regarded as “fair” acuity by Gittings and Fozard [16]), cup-to-disc ratio (>0.75), mean deviation (<−2 dB), PSD (>5 dB), and Roth error score (>128—in comparison, 126 was the maximum error score for the youngest adults in Erb et al. [17]). ERGs were also converted into dichotomous normal/abnormal values using site-specific lower bounds of normal from 9 age-matched control ERGs per site [18].

#### 3.4.3. Nonophthalmic Safety Evaluations

Physical exams and electrocardiograms were performed at baseline; dermatologic exams were performed at baseline and 6 months; blood pressure, pulse, and incidence of orthostatic hypotension were tested at all visits; and routine blood tests were performed at baseline and months 12 and 24. Treatment-emergent adverse events (AEs) were summarized per body system and drug.

### 3.5. Outcomes

#### 3.5.1. Prespecified

The primary outcome measure was presence or absence of COA change/deterioration from baseline to 2 years, adjudicated by the Expert Panel. Secondary outcomes included 1-year COA changes and prespecified 2-year subgroup analyses of COA changes, efficacy (UPDRS parts II+III total; Hoehn and Yahr), and nonophthalmic safety profile.

#### 3.5.2. Additional ERG Analysis

An FDA-required reanalysis evaluated treatment effects on the differences between the 2-year and baseline log_10_ values of each ERG parameter. Change scores for both eyes were averaged per visit per patient (assuming bilateral systemic effects). Frequency distributions of changes were plotted and compared between the 2 drugs.

#### 3.5.3. Exploratory Post Hoc

Additional exploratory post hoc analyses characterized baseline, 1-year, and 2-year ophthalmologic data, as described below.

### 3.6. Statistical Analyses

The FDA agreed to an empirical minimum sample size of 100 subjects/group completing ≥12 months' treatment. Recruitment goal was 300 subjects. Descriptive statistics were applied to primary and secondary results.

Cross-sectional baseline analyses modelled continuous outcomes by univariate and multivariate linear regression (acuity, cup/disk ratio horizontal and vertical, refraction spherical and cylindrical, IOP, mean deviation, pattern standard deviation, Roth error score, axis, and ERG parameters) for left and right eyes separately. Linear mixed all-eyes models included random effects for subjects with a compound symmetry correlation structure to account for within-subject dependency.

Logistic regression for dichotomous outcomes modelled abnormal response probability for left and right eyes separately. A generalized linear mixed model combined left- and right-eye data. Random effects for subjects were included in the logistic models, assuming a compound symmetry correlation structure. Covariates were age, sex, Hoehn–Yahr stage, and disease duration. Age and disease duration were categorized if empirical logit plots evidenced nonlinearity. Goodness-of-fit was assessed using the Hosmer–Lemeshow test. Odds ratios, 95% confidence intervals (CIs), and* P* values were derived for each model and covariate.

Longitudinal (on-treatment) analysis assessed ophthalmologic characteristics from baseline to 2 years similarly to the baseline assessment, but including drug assignment as a covariate and including baseline values as predictors of outcomes' change from baseline. Changes over 2 years were modelled against PD severity (baseline UPDRS parts II+III total); ERG amplitude changes from baseline were analyzed on a log_10_ scale.

All model assumptions were carefully monitored; remedial measures were implemented if any deviations were detected. Only results from all-eyes models are reported (left- or right-only models had similar results).

## 4. Results

### 4.1. Patients' Characteristics

#### 4.1.1. Demographics and PD Characteristics

**Figure 1:**
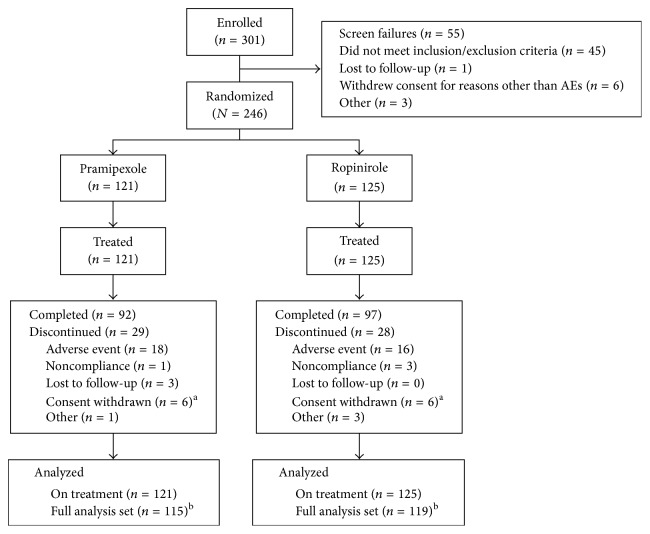
Subject disposition. Among the 55 patients who failed screening, 45 did not meet inclusion/exclusion criteria, 1 was lost to follow-up, 6 withdrew consent (for reasons not involving adverse events), and 3 had other reasons. ^a^For reasons other than adverse events. ^b^The FAS includes all patients from the treated set who had a baseline and at least 1 postbaseline comprehensive ophthalmology assessment (COA). AE: adverse event and FA: full analysis set.

Treated patients (*N* = 246; 157 men, 89 women) were 35–80 years of age (Table 1 and Figure 1). Baseline MMSE scores averaged 29.7 ± 1.2. Ethnicities were 95% Caucasian, 4% African American, and 1% Asian. Mean time since PD diagnosis was 1.1 years, maximum 9.4 years. Baseline UPDRS sum of parts II and III scores (mean ± SD and range) in the treated set pramipexole group (*n* = 121) was 28.8 ± 11.6, 3.0–66.0; for the ropinirole group (*n* = 125) 31.9 ± 13.4, 9.0–69.0) (Table 1). Baseline Hoehn–Yahr scores (Table 1) were stage 2 in 58.7% of pramipexole patients (71/121) and 56.8% of ropinirole patients (71/125). No one was in Hoehn–Yahr stage 0 at baseline.

#### 4.1.2. Treatment Parameters

During the maintenance phase, 113 pramipexole recipients received a mean ± SD daily dose of 3.00 ± 1.21 mg, median 3 mg; 116 ropinirole recipients received a mean ± SD daily dose of 9.57 ± 5.21 mg, median 9.00 mg.

Prestudy antiparkinsonian therapies (1 month before study drug start) continuing on-study in ≥2 patients were levodopa or derivatives in 13 patients total (carbidopa/levodopa, 3/121 pramipexole, 2.5%; 10/125 ropinirole, 8.0%), monoamine oxidase inhibitors in 32 patients (15/121 pramipexole, 12.4%; 17/125 ropinirole, 13.6%), amantadine in 18 patients (6/121 pramipexole, 5.0%; 12/125 ropinirole, 9.6%), tertiary amines (trihexyphenidyl HCl or biperiden HCl) in 7 patients (4/121 pramipexole, 3.3%; 3/125 ropinirole, 2.4%), and benzatropine in 2 patients (1 each [0.8%] per group).

Concomitant antiparkinsonian therapies started on-study in ≥2 patients were levodopa or derivatives in 85 patients (carbidopa/levodopa 33/121 pramipexole, 27.3%; 52/125 ropinirole, 41.6%; carbidopa/entacapone/levodopa 2/121 pramipexole, 1.7%; 5/125 ropinirole, 4.0%), monoamine oxidase inhibitors in 34 patients (12/121 pramipexole, 9.9%; 22/125 ropinirole, 17.6%), amantadine in 31 patients (13/121 pramipexole, 10.7%; 18/125 ropinirole, 14.4%), tertiary amines (trihexyphenidyl HCl, procyclidine HCl, or trihexyphenidyl) in 10 patients (2/121 pramipexole, 1.7%; 8/125 ropinirole, 6.4%), and rotigotine in 2 pramipexole patients only (1.7%). All nondrug therapies in ≥2 patients were in ropinirole recipients: physiotherapy in 5, acupuncture in 2, and speech rehabilitation in 2.

### 4.2. Baseline Ophthalmologic Findings

Baseline best-corrected ETDRS acuity averaged 56 ± 6 letters (approximately 20/20 Snellen). To facilitate comparisons, we dichotomized continuous variables using clinically established normal cutoff values shown in Table 2 along with prevalence of baseline eye abnormalities.

At baseline, the NORDIC Fundus Photography Reading Center of the University of Rochester (Rochester, NY) graded 30.0% of patients as having retinal hypopigmentation in ≥1 field, unrelated to geographic atrophy, 47.4% as having drusen, and 6.5% as having retinal hemorrhage or microaneurysm. No photographs contained hard exudates, retinal edema, or whitening.

Humphrey visual fields assessed by the Visual Field Reading Center were abnormal for mean deviation in 40.2% of patients and pattern standard deviation was abnormal in 9.0% of patients.

Baseline ERG measures (Table 2) showed that >33.3% of patients had abnormal mixed rod-cone b-wave implicit times and 24.4% abnormal cone flicker amplitudes.

#### 4.2.1. Exploratory Baseline Analyses

Age significantly predicted 19/81 baseline ophthalmologic outcomes; sex predicted only 3/81 outcomes. Hoehn–Yahr stage was associated with visual acuity, horizontal and vertical cup-to-disc ratios, motility smooth pursuit, and single flash cone response amplitude a- and b-waves. PD duration predicted lens condition, overall fundus photo clinical opinion, retinal hemorrhage or microaneurysm in inferior/superior nasal fields, RPE hyperpigmentation in the macula-centered field, and rod-cone mixed response implicit time b-wave. Effect sizes were generally small and deemed not clinically meaningful (Table 3).

### 4.3. Prespecified Posttreatment Outcomes

#### 4.3.1. Comprehensive Ophthalmologic Assessment (COA)

Expert Panel evaluations for the 2-year posttreatment COA (primary outcome) and the 1-year COA (secondary outcome) are shown in Table 4. Percentages of patients with 2-year COA deterioration, including those deemed clinically meaningful, did not differ significantly between drugs. No clinically meaningful ophthalmologic changes were deemed probably or definitely drug-related. Prespecified subgroup analyses (Table 5) were descriptive only.

#### 4.3.2. Efficacy Outcomes

Hoehn–Yahr stage distributions were similar between baseline and years 1 and 2. One subject per group transitioned from stage 1 to stage 0 after 1 year. Stage 2 proportions were 57.72% at baseline, 59.23% at year 1 and 61.62% at year 2. At year 2, 61.11% of patients remained in their starting Hoehn–Yahr stage; 10.10% improved (decreased) by 1 stage; 10.61% worsened (increased) by 1 stage; 5.56% improved by 2 stages, while 10.10% worsened by 2 stages. Only 0.51% improved by 3 stages and 2.02% worsened by 3 stages. The actual Hoehn–Yahr stage distributions at year 2 for, respectively, pramipexole and ropinirole were stage 0, 0.8% (1/121) and 0.0% (0/125); stage 1, 22.3% (27/121) and 18.4% (23/125); stage 1.5, 5.0% (6/121) and 9.6% (12/125); stage 2, 61.2% (74/121) and 58.4% (73/125); stage 2.5, 8.3% (10/121) and 8.0% (10/125); stage 3, 2.5% (3/121) and 5.6% (7/125).

UPDRS parts II+III sum scores improved in both groups after 1 year (mean changes from baseline: pramipexole −4.1; ropinirole −3.7) and 2 years (pramipexole −0.7; ropinirole −1.7).

#### 4.3.3. Nonophthalmologic Safety Outcomes

Adverse events are summarized in Table 6. Proportions with AEs or serious AEs (SAEs) were similar between groups. More ropinirole than pramipexole recipients had ≥1 severe or drug-related AE. There were no clinically significant changes in laboratory values.

### 4.4. Additional ERG Analysis

In the FDA-requested additional ERG analysis, the plotted frequency distributions of log_10_ change values between baseline and final treatment visits were similar for pramipexole and ropinirole.

### 4.5. Exploratory Post Hoc Longitudinal Analyses

The prevalence of RPE hypopigmentation on fundus photographs was unchanged from baseline to 2 years (30% baseline; 28.9% at 2 years). The percent of patients with drusen increased from 47.4% at baseline to 58.2% at 2 years.

Longitudinal multivariate models probed relationships of baseline PD severity (UPDRS) with ERG changes over 2 years on treatment (Table 7). Adjustment for baseline ophthalmologic outcomes in these models was essential, as they were the strongest predictors of 2-year changes in their outcomes.

## 5. Discussion

### 5.1. Interpretation of Prespecified Analysis Results

Retinal deterioration over 2 years' treatment in subjects with PD did not differ between pramipexole and ropinirole, as assessed by masked independent Expert Panel COA review. Modified Hoehn–Yahr stages and UPDRS II+III scores indicated therapeutic effects of study drug dosages given for PD signs and symptoms. The drugs had similar incidences of overall AEs and SAEs and individual AEs; safety profiles were consistent with labeling.

#### 5.1.1. Study Limitations

Protocol violations among treated subjects included refractive errors exceeding −6 D (with waivers) in 2 pramipexole and 2 ropinirole patients, bilateral abnormal ERG and retinal infarction in 1 pramipexole patient each, macular degeneration and retinal hemorrhage in 1 ropinirole patient each, diabetes mellitus in 1 pramipexole and 1 ropinirole patient, orthostatic hypertension in 1 pramipexole and 1 ropinirole patient, and malignant melanoma in 1 ropinirole patient.

Noting the limitations of statistical power and nonplacebo design, the resulting FDA-approved pramipexole IR label revision [9] stated, “There was no statistical difference in retinal deterioration between the treatment arms; however, the study was only capable of detecting a very large difference between treatments. In addition, because the study did not include an untreated comparison group (placebo treated), it is unknown whether the findings reported in patients treated with either drug are greater than the background rate in an aging population.”

#### 5.1.2. Generalizability

These findings may not be applicable to other dopamine agonists.

#### 5.1.3. Prespecified Analysis Conclusions

The COA results suggest no significant difference in risk for retinal deterioration between pramipexole and ropinirole in our study population. These results supported a revision of the Mirapex IR Prescribing Information approved by the FDA in March 2013, which remains in the most recent 2016 version [9].

### 5.2. Interpretation of FDA-Required Additional ERG Analysis

Frequency distributions of ERG log_10_ change values between baseline and final visits were similar for pramipexole and ropinirole, suggesting no differential effects on retinal electrophysiology. For both drugs, the general symmetry of the distribution reflected the variability in the test-retest reliability.

### 5.3. Interpretation of Baseline Findings and Additional Analyses

Our observation of baseline RPE hypopigmentation in 30% of patients was unexpected, as prior literature has not connected melanin losses with PD. After 2 years, RPE hypopigmentation prevalence was similar to baseline, 28.9%. We are not aware of previous clinical reports of RPE hypopigmentation in idiopathic PD, although PD perturbations of other retinal structures are documented (e.g., macular and retinal nerve fiber layer thinning [19] and inverse correlation of central minimum thickness with Hoehn–Yahr stage [20]). Retinal thickness distinguishes advanced PD patients from healthy persons [21]. Linear depigmenting RPE lesions occur in the Guam ALS/parkinsonism/dementia complex but are distinct from RPE hypopigmentation [22]. RPE hypopigmentation may be pathophysiologically related to PD because RPE cells produce dopamine [23]. Experimental chimeric loss of the retinal cell survival factor Ranbp in mice induced both RPE hypopigmentation and juvenile parkinsonism [24].

Drusen baseline frequency in our patients (47.4%) was generally similar to reported frequencies in geographically diverse studies in similar age groups [25–28].

Among other ERG findings, 33.3% of patients had abnormal rod-cone mixed response b-wave implicit times, 26.5% had abnormal single flash cone response a-wave implicit times, and 22.4% had abnormal cone flicker response amplitudes. Baseline UPDRS score was also a multivariate predictor of 2-year change in implicit time b-wave rod-cone mixed response in our patients. Retinal dopamine may participate in cone flash responsiveness [29] and rod signaling [30, 31]. A previous ERG study in treatment-naïve PD patients observed “subtle increase in the latency of their short-wavelength sensitive cone response” similar to our implicit time results; conversely, levodopa-experienced patients had worse ERG responses in levodopa withdrawal, improving after intravenous levodopa [29].

Age was a multivariate predictor of these baseline continuous parameters: baseline acuity, echoing the Beaver Dam Eye Study's significant acuity decline between the 43–52-year and ≥75-year age groups [32], spheroid, ERG amplitudes of cone flicker and b-wave rod responses and oscillatory potential, ERG a-wave, rod-cone mixed response implicit time (age effect also seen in [33]), Roth axis (Erb et al. cite increasing blue-yellow axis errors in seniors [17]), mean deviation [34], and pattern standard deviation. Age predicted these dichotomous parameters: abnormalities of cornea, lens, vitreous body, retinal hemorrhage or microaneurysm (stereo fields 3 and 7), RPE hyperpigmentation (stereo fields 5 and 7), visual field (reflecting published [35, 36] but not universally reported [37] age patterns), dichotomized Roth error score and axis [17].

Baseline Roth scores were predicted only by age, which is not surprising in view of reported increases in Roth error rates with age [17]; however, 2-year Roth score change was significantly associated only with baseline Roth score. A different color test yielded significantly higher error scores in PD subjects than controls, and error correlated with disease severity [38, 39], although another study found color errors in only 3 of 14 PD patients [40].

Age was a multivariate predictor of 2-year change in log_10_ oscillatory potential, consonant with general ERG age patterns [33]. Gender was a multivariate predictor of 2-year change in log_10_ cone flicker response and in Roth error score (the latter as in [17]). Other ERG 2-year changes were predicted only by their baseline values.

#### 5.3.1. Limitations and Generalizability of Additional Analyses

These results represent baseline ophthalmology findings and their longitudinal relationships only in the early idiopathic PD patients qualifying for inclusion.

## 6. Conclusions

The prespecified primary outcome, COA deterioration from baseline to 2 years scored by a masked Expert Panel, indicated no significant difference in risk for retinal deterioration in subjects with early idiopathic PD treated with pramipexole compared with ropinirole.

This study's finding of baseline RPE hypopigmentation was not previously reported in early PD and merits further study. Older age and more advanced Hoehn–Yahr stage significantly predicted lower visual acuity. Baseline ERG values and to some degree age significantly predicted most ERG changes at 2 years. Baseline Roth error score significantly predicted 2-year change in Roth error score, as did gender. Extensive ophthalmologic evaluation in this prospective cohort of early idiopathic PD patients suggests a possible relation of aging and PD to the observed ophthalmologic findings and could be further evaluated in patients with PD and in age-matched controls.

## Figures and Tables

**Table 1:** Demographic baseline characteristics of the 246 randomized and treated subjects who had ophthalmologic data.

	Randomized and treated set (*n* = 246)	Pramipexole (*n* = 121)	Ropinirole (*n* = 125)
Age in years, mean ± SD	58.3 ± 9.0	57.5 ± 9.3	59.1 ± 8.7
Range	35–80	35–78	36–80
Age group, *n* (%)			
<50	40 (16.3)	23 (19.0)	17 (13.6)
50 to <65	145 (58.9)	71 (58.7)	74 (59.2)
65 to <75	51 (20.7)	21 (17.4)	30 (24.0)
≥75	10 (4.1)	6 (5.0)	4 (3.2)
Men, *n *(%)	157 (63.8)	79 (65.3)	78 (62.4)
Women, *n* (%)	89 (36.2)	42 (34.7)	47 (37.6)
Race, *n* (%)			
Asian	2 (0.8)	1 (0.8)	1 (0.8)
African American	10 (4.1)	7 (5.8)	3 (2.4)
Caucasian	234 (95.1)	113 (93.4)	121 (96.8)
Time in years since PD diagnosis, mean ± SD	1.13 ± 1.46	0.97 ± 1.15	1.29 ± 1.70
Median	0.51	0.46	0.58
Range	0.0–9.4	0.0–5.2	0.0–9.4
Hoehn–Yahr stage, *n* (%)			
0	0	0	0
1	51 (20.7)	28 (23.1)	23 (18.4)
1.5	30 (12.2)	11 (9.1)	19 (15.2)
2	142 (57.7)	71 (58.7)	71 (56.8)
2.5	13 (5.3)	8 (6.6)	5 (4.0)
3	10 (4.1)	3 (2.5)	7 (5.6)
4	0	0	0
5	0	0	0
Sum of the UPDRS part II and part III total scores, mean ± SD	30.4 ± 12.7	28.8 ± 11.6	31.9 ± 13.4
Alcohol history, *n* (%)			
Does not drink	95 (38.6)	46 (38.0)	49 (39.2)
Number of patients with average consumption (not regarded as problematic by investigators)	151 (61.4)	75 (62.0)	76 (60.8)
Fulfils criteria for abuse/dependence	0	0	0
Most common concomitant diagnoses (>10%), *n* (%)			
Hypertension	93 (37.8)	42 (34.7)	51 (40.8)
Depression	74 (30.1)	38 (31.4)	36 (28.8)
Constipation	56 (22.8)	23 (19.0)	33 (26.4)
Drug hypersensitivity	56 (22.8)	25 (20.7)	31 (24.8)
Hypercholesterolemia	49 (19.9)	27 (22.3)	22 (17.6)
Anxiety	48 (19.5)	20 (16.5)	28 (22.4)
Back pain	48 (19.5)	26 (21.5)	22 (17.6)
Gastroesophageal reflux disease	37 (15.0)	19 (15.7)	18 (14.4)
Arthralgia	36 (14.6)	15 (12.4)	21 (16.8)
Insomnia	36 (14.6)	16 (13.2)	20 (16.0)
Headache	32 (13.0)	13 (10.7)	19 (15.2)
Osteoarthritis	30 (12.2)	11 (9.1)	19 (15.2)
Erectile dysfunction	29 of 157 men (18.5)	11 of 79 men (13.9)	18 of 78 men (23.1)
Pollakiuria	28 (11.4)	9 (7.4)	19 (15.2)
Arthritis	25 (10.2)	15 (12.4)	10 (8.0)
Hyperlipidemia	25 (10.2)	9 (7.4)	16 (12.8)

PD: Parkinson's disease; SD: standard deviation; UPDRS: Unified Parkinson's Disease Rating Scale.

**Table 2:** Percentages of patients (*N* = 246) with abnormal ophthalmologic or ERG values at baseline (see text of baseline cross-sectional analyses). Normal cutoff values for non-ERG parameters are shown; patients' ERG parameter abnormalities were scored against site-specific lower bounds of normal determined from 9 healthy control subjects per site. The Roth error score threshold for abnormality used in baseline analyses was lower than the Expert Panel's threshold used for the on-treatment COA.

Measure	Normal cutoff value (if applicable)	% of patients abnormal
Drusen, photography	Present	47.4
Mean deviation	<−2 dB	40.2
Lens	Abnormal	31.6
RPE hypopigmentation, photography	Present	30.0
Roth error score	128	18.8
RPE alterations, macular exam	Present	17.9
Lids	Abnormal	15.9
Snellen acuity	20/25	12.6
Cornea	Abnormal	10.6
Vitreous body	Present	9.8
Pattern standard deviation	>5 dB	9.0
Optic disc	Abnormal	8.9
Conjunctiva	Abnormal	8.1
Retinal hemorrhage or microaneurysm, photography	Present	6.5
Cylinder	2 diopters	6.1
Motility smooth pursuit	Abnormal	5.7
Retinal vessels	Abnormal	5.7
Nystagmus	Present	3.7
Cup-to-disc ratio, vertical	>0.75	2.4
Intraocular pressure	>21 mmHg	2.0
Cup-to-disc ratio, horizontal	>0.75	1.6
Motility eye position	Abnormal	1.6
Motility full versions	Abnormal	1.6
Iris	Abnormal	1.2
Sphere	<−5 or >5 diopters	0.4
Pupil consensuality	Abnormal	0.0
Direct response to light	Abnormal	0.0
Relative afferent pupillary defect	Abnormal	0.0
Hard exudates, photography	Present	0.0
Retinal edema or whitening, photography	Present	0.0

ERG Parameters	% of patients abnormal (by site-specific lower bounds of normal)	

Implicit time b-wave, rod-cone mixed response	33.3	
Implicit time a-wave, single flash cone response	26.5	
Amplitudes, cone flicker response	24.4	
Implicit time a-wave, rod-cone mixed response	22.6	
Amplitudes, oscillatory potentials	21.4	
Implicit time b-wave, rod-response	20.5	
Amplitude b-wave, single flash cone response	18.8	
Amplitude b-wave, rod-response	18.4	
Implicit time, cone flicker response	17.1	
Amplitude a-wave, single flash cone response	16.2	
Implicit time b-wave, single flash cone response	16.2	
Amplitude b-wave, rod-cone mixed response	15.8	
Amplitude a-wave, rod-cone mixed response	11.5	

COA: comprehensive ophthalmologic assessment; RPE: retinal pigment epithelium.

**Table 3:** Statistically significant multivariate predictors of baseline ophthalmologic outcomes (all-eyes models as described in Statistical Analyses).

Parameter	Predictor	Estimate	95% CI	*P *value
Number of letters correct	Age	−0.1817	−0.2487 to −0.1146	<0.00001
	Hoehn–Yahr stage 2 versus 1	−1.6445	−3.1788 to −0.1102	0.03577
Cup-to-disc ratio vertical	Hoehn–Yahr stage 1.5 versus 1	0.1258	0.03915–0.2125	0.00462
Cup-to-disc ratio horizontal	Hoehn–Yahr stage 1.5 versus 1	0.1162	0.03253–0.1999	0.00670
Spheroid	Age	0.04223	0.01230−0.07215	0.00588
ERG amplitude a-wave single flash cone response	Hoehn–Yahr stage 2.5 versus 1	17.6000	2.4890–32.7110	0.02264
ERG amplitude b-wave single flash cone response	Hoehn–Yahr stage 2.5 versus 1	35.4344	6.5034–64.3654	0.01659
	Hoehn–Yahr stage 3 versus 1	32.6769	0.2069–65.1470	0.04857
ERG amplitude cone flicker response	Age	−0.6556	−1.1121 to −0.1991	0.00507
	Sex	−9.5579	−18.0134 to −1.1023	0.02690
ERG amplitude b-wave rod response	Age	−1.0889	−2.1294 to −0.04848	0.04032
ERG implicit time b-wave rod-cone mixed response	PD duration	1.9444	0.9465–2.9423	0.00016
ERG implicit time a-wave rod-cone mixed response	Age	0.08605	0.03841−0.1337	0.00045
ERG amplitudes—oscillatory potentials	Age	−0.9213	−1.4816 to −0.3611	0.00137
Roth axis	Age	0.8871	0.1830–1.5912	0.01376
Mean deviation	Age	−0.05078	−0.08477 to −0.01680	0.00356
Pattern standard deviation	Age	0.02940	0.003796–0.05501	0.02460

Dichotomous parameters	Predictor	Odds ratio	95% CI	*P* value

Motility—smooth pursuit	Hoehn–Yahr stage high versus low	7.54365	1.1907–47.7944	0.03207
Cornea	Age	1.09296	1.0390–1.1497	0.00064
Lens	Age	1.08008	1.0442–1.1172	0.00001
	PD duration	0.77566	0.6209–0.9690	0.02545
Vitreous body	Age	1.09915	1.0400–1.1616	0.00088
	Male versus female	0.35561	0.1435–0.8811	0.02569
Overall clinical opinion-fundus photo	PD duration	0.79028	0.6368–0.9808	0.03283
Retinal hemorrhage or microaneurysm—stereo field 3—temporal to macula	Age	1.12277	1.0203–1.2355	0.01794
Retinal hemorrhage or microaneurysm—stereo field 6—superior nasal	PD duration	1.89892	1.1436–3.1530	0.01340
Retinal hemorrhage or microaneurysm—stereo field 7 inferior nasal	Age	1.17771	1.0662–1.3009	0.00137
	PD duration	1.57130	1.1120–2.2204	0.01064
RPE hyperpigmentation stereo field 2 macula centered	PD duration	1.28670	1.0752–1.5398	0.00613
RPE hyperpigmentation stereo field 5—inferior temporal	Age	1.04950	1.0011–1.1002	0.04483
RPE hyperpigmentation stereo field 7—inferior nasal	Age	1.04595	1.002–1.0938	0.04906
Visual field	Age	1.04813	1.0185–1.0786	0.00141
Dichotomized Roth error score^a^	Age	1.04722	1.0194–1.0759	0.00088
Dichotomized Roth axis^b^	Age	1.02231	1.0002–1.0449	0.04753

^a^For this analysis Roth error scores were dichotomized at the median value of 12, which was also the published minimum score for the youngest adult subjects in the study of Erb et al. 1998 [17].

^b^For this analysis Roth axis scores were dichotomized at the median value of 45.

CI: confidence interval; PD: Parkinson's disease; RPE: retinal pigment epithelium.

**Table 4:** Summary of Expert Panel's COA (full analysis set, LOCF). The Expert Panel defined worsening as any of the following (thresholds determined from clinical experience and reading centers' assessment of control subjects): Roth 28 error scores >295 right eye, >271 left; MD change of −2 dB confirmed by a later test; field defect clusters of 3 locations at *P* < 0.05 or 2 at *P* < 0.01; ERG parameter change scores (log_10_ differences from log_10_ baseline values) <2.56 times standard deviation of repeatability; fundus changes from “absent” to “obvious” for any finding; acuity loss ≥10 letters; pupil change from normal to abnormal; IOP >22 mmHg. Prespecified subgroup analyses of the COA results are shown in Table 5.

Parameters	After 2 years on treatment^a^		After 1 year on treatment^b^	
	Pramipexole (*n* = 115)	Ropinirole (*n* = 119)	Pramipexole (*n* = 115)	Ropinirole (*n* = 119)
Worse from baseline^c,d^, *n* (%)	34 (29.6)	33 (27.7)	28 (24.3)	21 (17.6)
Estimated RR for pramipexole compared with ropinirole	1.07 (95% CI 0.71, 1.60)		Not calculated for 1-year data	
Number with clinically significant ophthalmologic change^e,f^, *n* (%)	17 (14.8)	20 (16.8)	14 (12.2)	15 (12.6)
Number of subjects with study drug-related change, *n* (%)				
Definitely not	1 (0.9)	4 (3.4)	1 (0.9)	2 (1.7)
Unlikely	6 (5.2)	6 (5.0)	5 (4.3)	6 (5.0)
Possibly	10 (8.7)	10 (8.4)	8 (7.0)	7 (5.9)
Probably	0	0	0	0
Definitely	0	0	0	0

^a^18 subjects per group had responses carried forward.

^b^15 subjects had responses carried forward.

^c^Eight subjects were assessed as “worse from baseline” in 2-year data based on responses carried forward (2 subjects in pramipexole group and 6 in ropinirole group).

^d^Four subjects were assessed as “worse from baseline” in 1-year data based on responses carried forward (1 subject in pramipexole group and 3 subjects in ropinirole group).

^e^There were 2 subjects for whom the Expert Panel could not assess clinical significance due to unreliable visual field testing in 2-year data.

^f^The Expert Panel was able to assess clinical significance for all subjects in 1-year data.

CI: confidence interval; COA: comprehensive ophthalmology assessment; IOP: intraocular pressure; LOCF: last observation carried forward; MD: mean deviation; RR: relative risk.

**Table 5:** Subgroup analysis: summary of Expert Panel's comprehensive ophthalmology assessment (COA) following 2 years on study drug (full analysis set, last observation carried forward [LOCF]). Shown are numbers of patients in each subgroup assessed to have worsening from baseline on the COA, as defined in the legend of Table 4/number of patients in each subgroup within each treatment arm.

	Pramipexole (*n* = 115)	Ropinirole (*n* = 119)
Number worse from baseline^a^, *n* (%)	34 (29.6)	33 (27.7)

Gender, *n* (%)		
Male	24/74 (32.4)	18/77 (23.4)
Female	10/41 (24.4)	15/42 (35.7)
Race, *n* (%)		
White	32/107 (29.9)	32/115 (27.8)
Nonwhite	2/8 (25.0)	1/4 (25.0)
Age in years, *n* (%) (age grouping 1)		
<65	23/91 (25.3)	25/88 (28.4)
≥65	11/24 (45.8)	8/31 (25.8)
Age in years, *n* (%) (Age Grouping 2)		
<50	4/22 (18.2)	6/17 (35.3)
50 to <65	19/69 (27.5)	19/71 (26.8)
65 to <75	7/19 (36.8)	5/27 (18.5)
≥75	4/5 (80.0)	3/4 (75.0)
Concomitant use of levodopa, *n* (%)		
No	25/82 (30.5)	16/68 (23.5)
Yes	9/33 (27.3)	17/51 (33.3)
Hoehn–Yahr stage at baseline, *n* (%)		
1	9/26 (34.6)	8/23 (34.8)
1.5	3/10 (30.0)	2/17 (11.8)
2	19/69 (27.5)	21/69 (30.4)
2.5	2/7 (28.6)	1/5 (20.0)
3	1/3 (33.3)	1/5 (20.0)

^a^Eight subjects were assessed as “worse from baseline” based on responses carried forward (2 subjects in the pramipexole group and 6 in the ropinirole group).

**Table 6:** Summary of adverse events (AEs; treatment-emergent irrespective of relationship to treatment, unless described specifically as treatment-related).

AEs	Pramipexole (*n* = 121)	Ropinirole (*n* = 125)
Any AE, *n* (%)	119 (98.3)	122 (97.6)
Severe AE, *n* (%)	24 (19.8)	35 (28.0)
Drug-related AE^a^, *n* (%)	105 (86.8)	113 (90.4)
Other significant AEs^b^, *n* (%)	62 (51.2)	64 (51.2)
AEs leading to discontinuation of study drug, *n* (%)	18 (14.9)	16 (12.8)
Serious AEs, *n* (%)	22 (18.2)	21 (16.8)

AEs occurring in ≥10% subjects in either group		

Gastrointestinal disorders	64 (52.9)	87 (69.6)
Constipation	18 (14.9)	26 (20.8)
Nausea	31 (25.6)	59 (47.2)
General disorders/administrative-site conditions	60 (49.6)	51 (40.8)
Fatigue	27 (22.3)	25 (20.0)
Peripheral edema	22 (18.2)	18 (14.4)
Infections and infestations	48 (39.7)	52 (41.6)
Nasopharyngitis	17 (14.0)	16 (12.8)
Upper respiratory tract infection	12 (9.9)	13 (10.4)
Musculoskeletal and connective tissue disorders	52 (43.0)	57 (45.6)
Back pain	18 (14.9)	9 (7.2)
Pain in extremity	12 (9.9)	13 (10.4)
Nervous system disorders	86 (71.1)	101 (80.8)
Dizziness	27 (22.3)	35 (28.0)
Headache	15 (12.4)	27 (21.6)
Somnolence	53 (43.8)	71 (56.8)
Sudden onset of sleep	11 (9.1)	16 (12.8)
Psychiatric disorders	50 (41.3)	72 (57.6)
Anxiety	10 (8.3)	14 (11.2)
Depression	8 (6.6)	15 (12.0)
Insomnia	19 (15.7)	27 (21.6)
Vascular disorders	26 (21.5)	31 (24.8)
Orthostatic hypotension	10 (8.3)	14 (11.2)
Drug-related AEs affecting >15% of patients		
Somnolence	50 (41.3)	69 (55.2)
Nausea	28 (23.1)	56 (44.8)
Fatigue	23 (19.0)	21 (16.8)
Dizziness	21 (17.4)	30 (24.0)
AEs of special interest		
Sudden onset of sleep	19 (16%)	29 (23%)
On-treatment evidence of melanoma	0 (0%)	1 (0.8%) (1 more patient reported melanoma as an SAE between visits)
Clinically relevant AEs (impulse control disorders)		
Binge eating	1 (0.8%)	0 (0%)
Dermatillomania	1 (0.8%)	0 (0%)
Eating disorder	1 (0.8%)	0 (0%)
Impulse control disorder	1 (0.8%)	0 (0%)
Impulsive behavior	1 (0.8%)	0 (0%)
Compulsions	0 (0%)	1 (0.8%)
Hypersexuality	0 (0%)	1 (0.8%)
Compulsive shopping	1 (0.8%)	1 (0.8%)
Pathologic gambling	1 (0.8%)	1 (0.8%)
Fatal SAE (nondrug related)	1 (0.8%): stab wound	0 (0%)

^a^As defined by the investigator.

^b^Marked hematological and other laboratory abnormalities (other than those meeting the definition of serious) and any events that led to an intervention, including withdrawal of test drug/investigational product treatment, dose reduction, or significant additional concomitant therapy, other than those reported as serious adverse events (SAE).

**Table 7:** Statistically significant (*P* < 0.05) multivariate predictors of 2-year change in ophthalmologic parameters and ERG amplitudes (log_10_ [ERG amplitude] difference from baseline to 2 years). Longitudinal models were developed for *all eyes *as described in Section 3.

Parameter	Predictor	Effect estimate	95% CI	*P *value
2-year change in IOP	Baseline IOP	−0.6763	−0.7682 to −0.5845	<0.00001
2-year change in log_10_ oscillatory potential (OP)	Age	−0.00452	−0.00760 to −0.00144	0.00423
	Baseline OP	−0.6236	−0.7558 to −0.4915	<0.00001
2-year change in log_10_ cone flicker response	Baseline cone flicker response	−0.6382	−0.7327 to −0.5437	<0.00001
	Male versus female	−0.06067	−0.1080 to −0.01337	0.01220
2-year change in log_10_ a-wave single flash cone response	Baseline a-wave single flash cone response	−0.8217	−0.9050 to −0.7384	<0.00001
2-year change in log_10_ a-wave rod cone mixed response	Baseline a-wave rod cone mixed response	−0.6440	−0.7471 to −0.5408	<0.00001
2-year change in log_10_ b-wave rod cone mixed response	Baseline b-wave rod cone mixed response	−0.7409	−0.8399 to −0.6420	<0.00001
2-year change in log_10_ b-wave single flash cone response	Baseline b-wave single flash cone response	−0.6523	−0.7358 to −0.5688	<0.00001
2-year change in implicit time b-wave rod cone mixed response	Baseline implicit time b-wave rod cone mixed response	−0.8527	−0.9409 to −0.7646	<0.00001
	Baseline UPDRS	0.04867	0.01477 to 0.08258	0.00512
2-year change in Roth error score	Baseline Roth error score	−0.4791	−0.5489 to −0.4094	<0.00001
	Male versus female	20.6491	3.0181 to 38.2802	0.02194

No covariates significantly predicted 2-year change in spheroid.

CI: confidence interval; IOP: intraocular pressure; UPDRS: Unified Parkinson's Disease Rating Scale.
